# Volume electron microscopy: analyzing the lung

**DOI:** 10.1007/s00418-020-01916-3

**Published:** 2020-09-17

**Authors:** Jan Philipp Schneider, Jan Hegermann, Christoph Wrede

**Affiliations:** 1 Institute of Functional and Applied Anatomy, Hannover Medical School, 30625 Hannover, Germany; 2 Biomedical Research in Endstage and Obstructive Lung Disease Hannover (BREATH), Member of the German Center for Lung Research (DZL), 30625 Hannover, Germany; 3 Research Core Unit Electron Microscopy, Hannover Medical School, 30625 Hannover, Germany

**Keywords:** Lung, Serial sectioning transmission electron microscopy, Array tomography, Electron tomography, Focused ion beam scanning electron microscopy, Serial block-face scanning electron microscopy, Volume electron microscopy, 3D reconstruction

## Abstract

Since its entry into biomedical research in the first half of the twentieth century, electron microscopy has been a valuable tool for lung researchers to explore the lung’s delicate ultrastructure. Among others, it proved the existence of a continuous alveolar epithelium and demonstrated the surfactant lining layer. With the establishment of serial sectioning transmission electron microscopy, as the first “volume electron microscopic” technique, electron microscopy entered the third dimension and investigations of the lung’s three-dimensional ultrastructure became possible. Over the years, further techniques, ranging from electron tomography over serial block-face and focused ion beam scanning electron microscopy to array tomography became available. All techniques cover different volumes and resolutions, and, thus, different scientific questions. This review gives an overview of these techniques and their application in lung research, focusing on their fields of application and practical implementation. Furthermore, an introduction is given how the output raw data are processed and the final three-dimensional models can be generated.

## Introduction

Molecular oxygen is essential for our living organism and its aerobic metabolism. Among others, it is necessary for the production of adenosine triphosphate (ATP), the universal energy supply of cells, within in the scope of oxidative phosphorylation and the respiratory chain in mitochondria (see standard textbooks of biochemistry, e.g., Voet et al. 2013; Berg et al. 2019).

The incorporation of oxygen into the blood takes place in the lung. With the breathing air, it flows through the bronchial tree into the alveoli, small units at the end of the respiratory tree where the breathing air and the blood get into intimate contact, separated only by a very thin blood–air barrier, so that oxygen can diffuse into the blood. Likewise, carbon dioxide diffuses from the blood into the alveoli so that it can be exhaled. Adjacent alveoli are separated by thin alveolar septa. These septa house a dense capillary network (alveolar capillary network, ACN) for gas exchange that is fed by branches of the pulmonary arteries. The septa are lined by a single-layered epithelium consisting of vast, but very thin alveolar epithelial type 1 (AE1) cells and cuboidal alveolar epithelial type 2 (AE2) cells. On top of the epithelium, a fluid alveolar lining layer is found that contains the pulmonary surfactant, a lipid/protein composite provided by AE2 cells to reduce surface tension and prevent alveolar atelectasis. Within the interstitial compartment resident fibroblasts and contractile cells (e.g., myofibroblasts) as well as free interstitial cells serving host defense are found. A continuous network of elastic and collagenous fibers, spanning from the hilum to visceral pleura, forms a distensible fiber scaffold supporting the ACN (Ochs and Weibel 2008, the reader is referred to this article for more details).

For the sake of efficient gas exchange, the blood–air barrier (consisting of alveolar epithelium, capillary endothelium and the interstitial space in between) has to be kept thin (Ochs and Weibel 2008). This is accomplished by its clever ultrastructure: beyond the nucleus, endothelial and AE1 cells are reduced to thin cytoplasmic extensions that line the capillary and the alveolar surface, respectively (Ochs and Weibel 2008). In humans, the AE1 cell extensions are attenuated to a thickness of 0.1–0.2 µm (Low 1953; Weibel 1971) or even less (Dobbs et al. 2010). Additionally, the space between both cells may be reduced to a fused basement membrane (which is the case at about half of the capillary surface), resulting in minimal barrier thickness of 0.2–0.4 µm in humans (Gehr et al. 1978). This organization makes some demands on structural investigations of the alveolar septa. Considering the blood–air barrier and its constituents, we find ourselves at or even beyond the limit of conventional light microscopic resolution, which is ~ 0.2 µm (Keller and Goldman 2006), so that microscopic techniques like electron microscopy (EM) with higher resolution are necessary. Indeed, it took until the middle of the twentieth century, that a continuous epithelial lining on alveolar septa could be proven by Frank Low (Low 1952, 1953) using EM (for historical review see, Ochs et al. 2016; West 2016).

EM, truly, opened a new era in lung research. Detailed structural investigations of the alveolar septum with deep impact on our current physiological understanding of the lung became available, driven amongst others by E. R. Weibel^†^ (at last Institute of Anatomy, University of Bern, Bern, Switzerland) and his colleagues. Amongst others, these comprise the demonstration of the extracellular surfactant lining layer (Weibel and Gil 1968), the demystification of Albert von Koelliker’s non-nucleated plates (Weibel 1971), the morphometric estimation of the pulmonary diffusion capacity (Gehr et al. 1978) and a detailed quantitative assessment of the human alveolar septal wall (Crapo et al. 1982).

Traditionally, conventional microscopy, however, is faced with the problem that thin sections of the tissue of interest have to be prepared, which disassembles its three-dimensional (3D) nature (for transmission electron microscopy (TEM), in general, 60–90 nm thin sections are used (Hoppert 2003)). Indeed, (single section) TEM can reveal fine structural details, such as Weibel–Palade bodies in the endothelium (Weibel 2012, 2017) or single lipid lamellae in lamellar bodies (LBs) of AE2 cells (Ochs 2010), but it may fail to transport important spatial information, like the belonging of two separate cell profiles to the same cell [examples are found in Schneider et al. (2019, 2020)].

Fortunately, ever since EM has found its entry into biomedical research, microscopic evolution has not yet come to an end. The microscopic field is advancing rapidly, which is not at last visible by the fact that the Nobel prize in 2014 was awarded to Eric Betzig, Stefan Hell and William E. Moerner “for the development of super-resolved fluorescence microscopy” (Möckl et al. 2014) and in 2017 to Jacques Dubochet, Joachim Frank and Richard Henderson for their contribution to the development of cryo-EM (Callaway 2017).

With respect to EM, the portfolio of established techniques applicable to the lung has been amplified greatly in the last decades (for review see, Ochs et al. 2016). In particular, techniques that restore the third dimension lost during sectioning became available. In other words: Today, we are not only able to visualize the individual constituents of the blood–air barrier, but also we are able to reconstruct them in 3D and put them into a broad topographic context, that offers new (more complete) insights into the structural relationships within the lung. Hereinafter, we will provide a detailed overview of these “volume electron microscopy” techniques, discuss their area of application and give examples of available studies. We will discuss the special demands of “lung volume electron microscopy”, how to cope with them, and we will exemplify how to convert the EM raw data into 3D models.

## Volume electron microscopic techniques and their applications

**Fig. 1:**
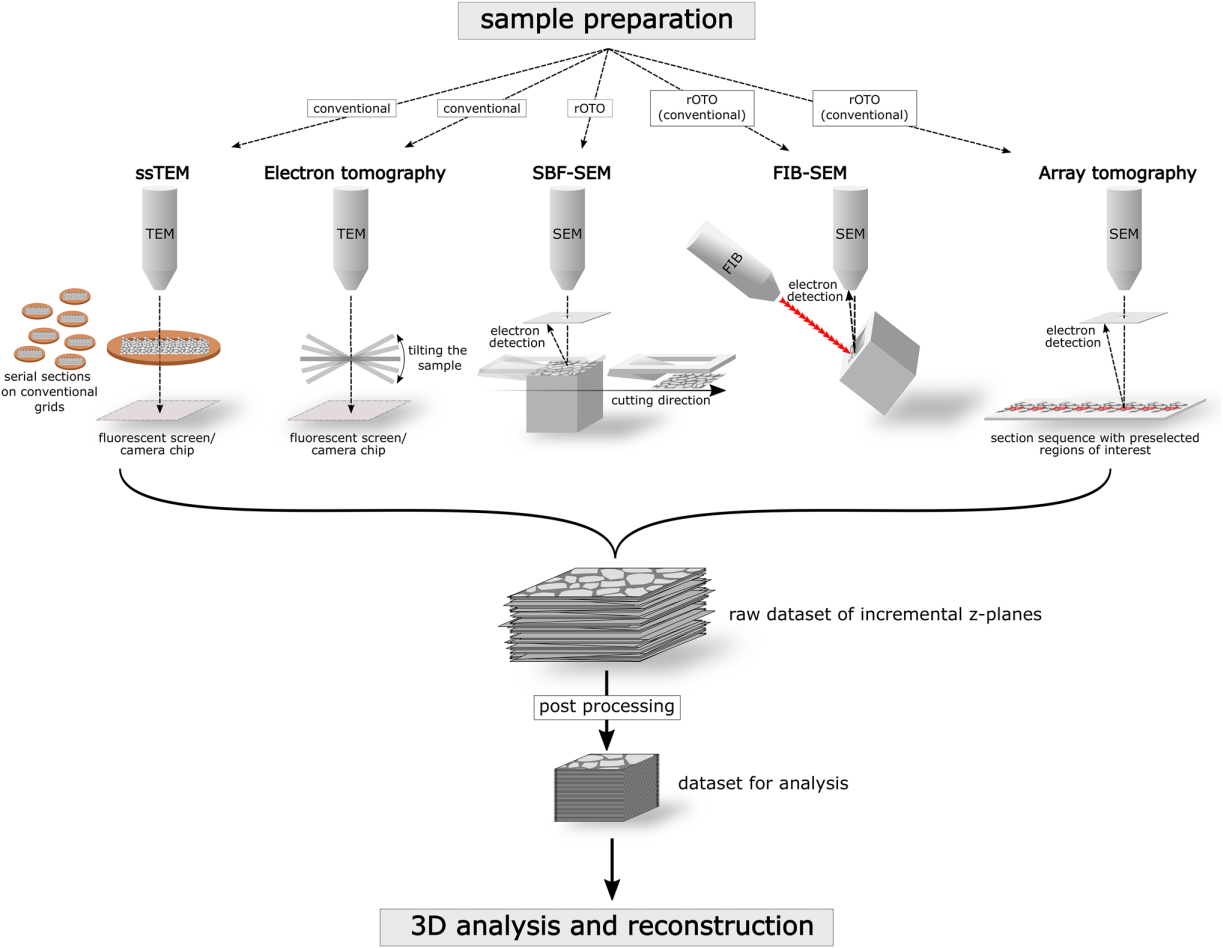
Overview of the different volume electron microscopic (EM) techniques: ssTEM (serial sectioning transmission electron microscopy), electron tomography, SBF-SEM (serial block-face scanning electron microscopy), FIB-SEM (focused ion beam scanning electron microscopy), array tomography. After appropriate sample preparation the sample is imaged and a raw dataset of incremental *z*-planes is generated. This dataset is post-processed (e.g., compression, filtering, alignment) to the final dataset for 3D analysis and reconstruction. For details see text

**Fig. 2:**
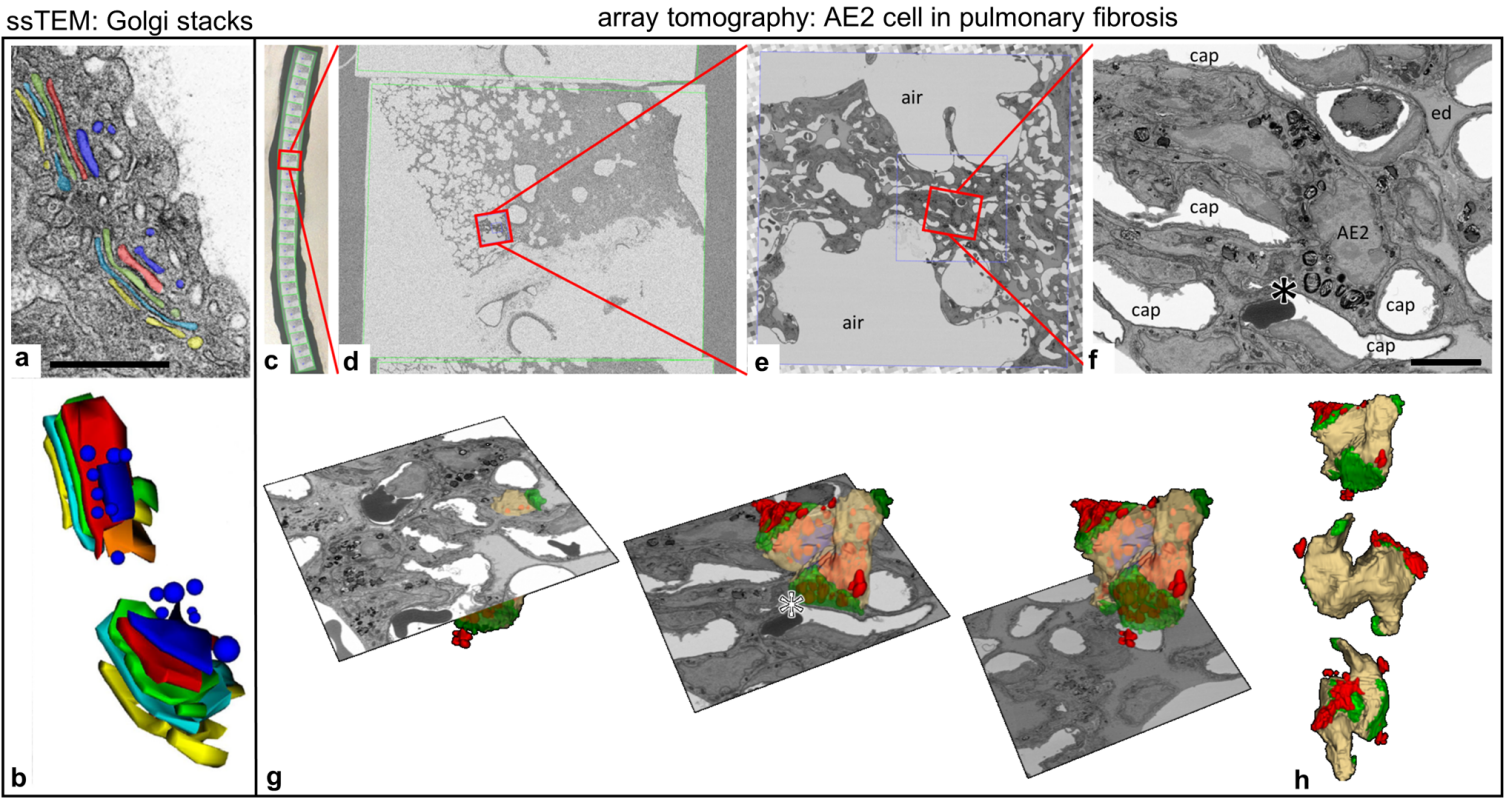
Serial sectioning TEM (ssTEM) and array tomography (AT). Left: ssTEM of Golgi stacks in an AE1 cell of a mouse lung; reconstructed from nine serial sections, recorded consecutively in a TEM. Cisternae of two Golgi stacks are segmented manually in different colors. **a** One electron micrograph of the series with the manual segmentation; **b** the resulting 3D model of the Golgi stacks; colors correspond to the segmentation shown in a [reused with permission from Hegermann et al. (2019); copyright/licensing remains there]. Scale bar: 500 nm. Right: AT of an AE2 cell in fibrotic mouse lung parenchyma. **c**–**f** Images of increasing magnification from a ribbon of sections (**c**) to an overview of a whole section (**d**) and an intermediate magnification (**e**) up to one AE2 cell (**f**, asterisk), which is trapped in fibrotic tissue (scale bar: 5 µm). **g** 3D reconstruction of the AE2 cell shown above, three individual section planes out of the recorded image stack are shown in relation to the 3D model. The model is shown in h in different rotations (color code: green: apical membrane; beige: basolateral membrane; red: extracellular surfactant); *cap* capillary, *ed* alveolar edema fluid. [Reused with permission from Beike et al. (2019); copyright/licensing remains there]

**Fig. 3:**
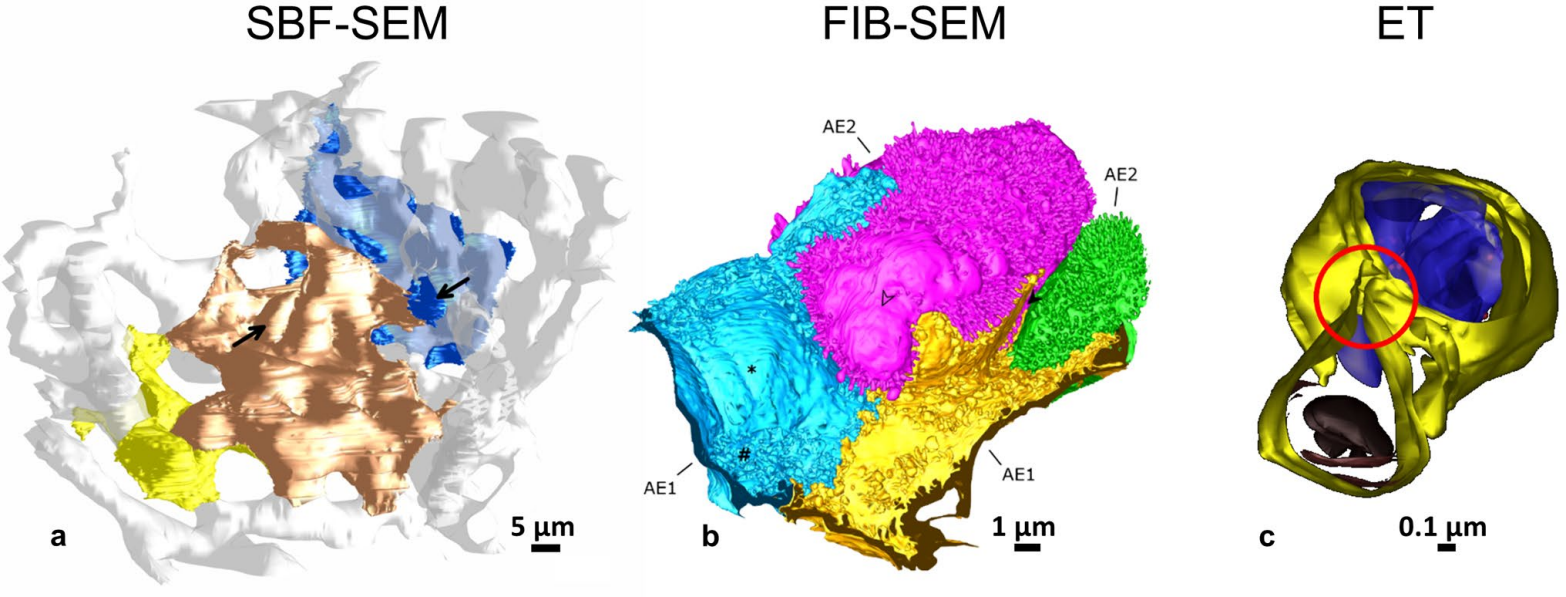
3D models gained by serial block-face scanning electron microscopy (SBF-SEM), focused ion beam scanning electron microscopy (FIB-SEM) and electron tomography (ET). **a** Reconstruction of human AE1 cells (yellow, gold, blue) and the alveolar capillary network (white) based on a SBF-SEM dataset. Arrows indicate the position of AE1 cell nuclei (adapted with permission of the American Thoracic Society from reference Schneider et al. (2019). Copyright © 2020 American Thoracic Society. All rights reserved. The American Journal of Respiratory and Critical Care Medicine is an official journal of the American Thoracic Society. Readers are encouraged to read the entire article for the correct context at https://www.atsjournals.org/doi/full/10.1164/rccm.201810-1866LE [accessed July 20th, 2020]. The authors, editors, and The American Thoracic Society are not responsible for errors or omissions in adaptations). **b** Reconstruction of an almost complete human AE2 cell (pink) with parts of adjacent AE1 cell domains (blue, yellow) and an additional AE2 cell (green) based on a FIB-SEM dataset. Note the high level of details, for example the cellular surface including the dense microvilli lawn on AE2 cells (reused with permission from Schneider et al. (2020); copyright/licensing remains there). **c** ET reconstruction of a lamellar body (top) and an autophagosome (bottom) inside an AE2 cell (mouse lung). Individual lipid membranes can be recognized, which in this case reveals a connection of the two organelles (red circle) (reused with permission from Mahavadi et al. (2015); copyright/licensing remains there)

**Fig. 4:**
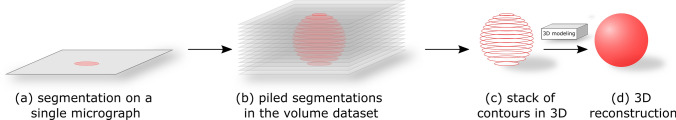
The figure depicts the 3D reconstruction of a spherical object in a stack of incremental *z*-planes. On a single micrograph, the profile(s) of the object of interest are delineated (**a**). This is repeated throughout the dataset until the entire object is captured (**b**). The pile of contours (**c**) is then utilized by the reconstruction software to model the object in 3D (**d**)

**Fig. 5:**
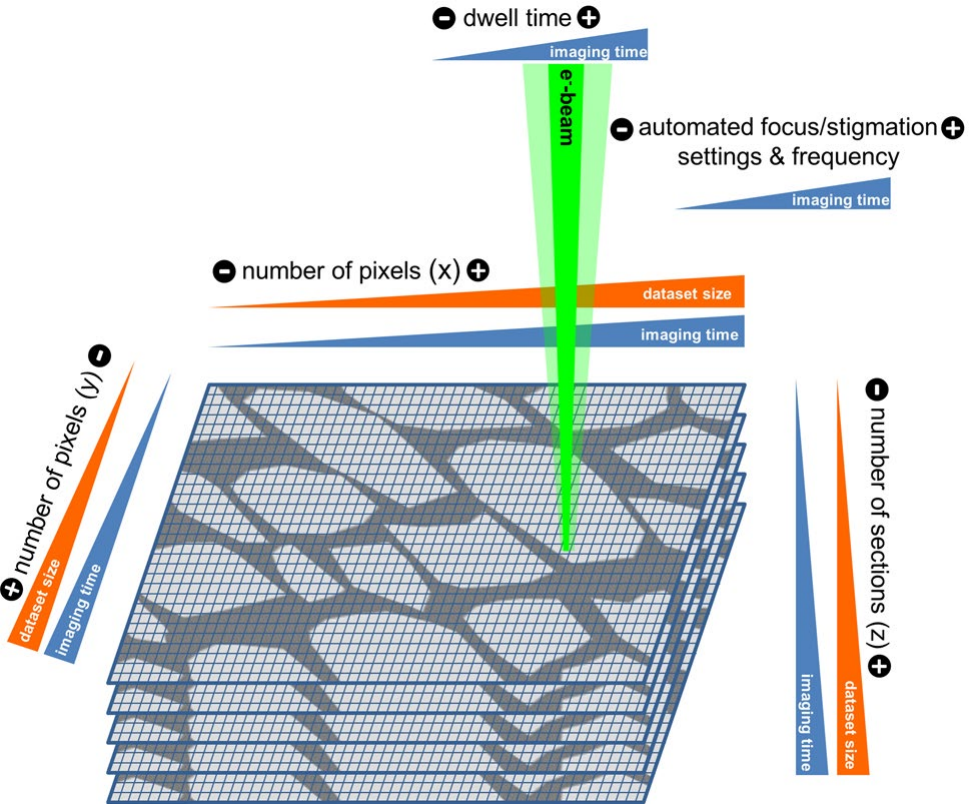
Significant imaging parameters for recording time and dataset size for SEM-based volume techniques (SBF-SEM, FIB-SEM, AT): higher number of pixels (*x*-, *y*-axis), more images/sections (*z*-axis), longer dwell time, more frequent automated focus and stigmation, number of pixels and dwell time used for focus/stigmation increase the total recording time. Number of pixels and number of images/sections determine the size of the dataset

Scientists have strived to gather 3D information from sections for more than 200 years now. Basic principles and methodologies have already been described at the end of the nineteenth century (for example, Strasser 1886): The 3D structure of an object can be seized by tracing and analyzing its boundaries on a sequence of parallel sections through the object. Simple imagination, drawings or modeling may then be used to gain a 3D conception. Outlines can either be transferred to a single plane which gives a contour map comparable to those in a geography atlas or the contours can be piled in correct scale to form a real 3D model. All methods introduced here rely on this basic principle of serial sections (physical or virtual). However, they differ in (1) the way how the stack of serial images is generated (cf. Borrett and Hughes 2016), (2) the size of the volume that can be analyzed and (3) the maximal *x*-, *y*- but also *z*-resolution. The latter two determine which method is most suitable for following a certain scientific question (cf., Müller-Reichert et al. 2010). Table 1 lists the advantages, disadvantages, volume sizes, resolutions and exemplary targets of the different methods reviewed in this article in lung research. A schematic overview is given in Fig. 1, where the different techniques are shown as schematic drawings, depicting their general principles, modes of operation and appropriate sample treatment. All methods provide a raw dataset of incremental *z*-planes, which have to be processed for 3D analysis and reconstructions. Figures 2 and 3 depict examples of resulting image data and models obtained by these methods in lung research, covering different sample sizes and resolutions. Figure 4 illustrates the general principle of 3D reconstructions based on incremental *z*-planes for all techniques. The variation of imaging parameters in the scanning electron microscopy (SEM)-based techniques and their impact on data size and recording time are illustrated in Fig. 5.

**Table 1:** Overview of advantages, disadvantages, volume sizes, resolutions and exemplary targets of different volume EM techniques

	Serial sectioning TEM	Electron tomography	Serial block-face SEM	Focused ion beam SEM	Array tomography
Advantages	Standard EM equipment sufficient, High lateral (*x*, *y*) TEM resolution	High TEM resolution in all dimensions	Automated acquisition of large volumes, no section based artifacts	Automated acquisition, best SEM-based resolution in all dimensions, little artifacts, applicable on (archive) samples embedded for TEM	Applicable without specially equipped SEMs, very versatile, allows imaging of large areas
Disadvantages	Needs experience in ultramicrotomy, time-consuming, risk of section losses and/or artifacts due to section handling	Limited to small volumes (*z*-dimension ~ 300 nm)	Special staining protocols needed, no post-staining and immuno-labeling on sections possible	Limited to small volumes (~ 20 µm edge length), expensive consumable supplies, no post-staining and immuno-labeling on sections possible	Needs experience in ultramicrotomy, time-consuming, risk of section losses and/or artifacts due to section handling (less difficulties than in ssTEM)
Dimensions	*x*, *y*: < 3 mm *z*: depending on effort	*x*, *y*: ~ 20 µm (more is possible but not reasonable) *z*: ~ 300 nm	*x*, *y*, *z*: ~ 500 µm	*x*, *y*, *z*: ~ 20 µm	*x*, *y*: ~ 3 mm *z*: depending on effort
Achievable resolutions* and examples of discernible structures	*x*, *y*: ~ 2 nm	*x*, *y*: ~ 2 nm	*x*, *y* ~ 15 nm	*x*, *y*: ~ 10 nm	*x*, *y*: ~ 10 nm
	*z*: ~ 60 nm	*z*: ~ 2 nm	*z*: ~ 50–80 nm	*z*: ~ 10 nm	*z*: ~ 60 nm
	Both phospholipid leaflets of unit membrane in *x*, *y*	Both phospholipid leaflets of unit membrane in all dimensions	Organelles and course of cell borders	Details of organelles	Comparable to FIB in *x*, *y*
Exemplary targets in lung research	Structures requiring high lateral (*x*, *y*) TEM resolution and delineation over a wide *z*-range (e.g., cell junctions)	Ultrastructural details like cisternae of Golgi apparatus, microtubules of cilia, cytoskeleton	Overall tissue architecture, e.g., context of alveoli and alveolar capillary network	One or few individual cells regarding inter- and intracellular details like microvilli, lamellar bodies, autophagy	Particular areas like fibrotic regions, specific cell types like alveolar macrophages

*Judged by what we see

## Serial sectioning transmission electron microscopy (ssTEM)

Serial sectioning transmission electron microscopy (ssTEM) is the pioneer among the 3D EM techniques and was already available in the 1950s (Gay and Anderson 1954, 1955; Bang and Bang 1957; Titze and Genoud 2016). Consecutive thin sections, prepared with a routine ultramicrotome are imaged with a conventional TEM and used for 3D analyses (for a detailed review, see Miranda et al. 2015).

This technique has already been applied in numerous studies of lung research covering various species and topics. The range of species includes, for example, the toad (Campbell et al. 1978; Rogers and Haller 1978, 1980), mouse (Hung et al. 1973), rat (Young et al. 1985; Burri and Tarek 1990; Weiss and Burri 1996), hamster (Pearsall et al. 1985), rabbit (Lauweryns and Cokelaere 1973; Walker et al. 1995), cat (Mercurio and Rhodin 1976, 1978, 1984), dog (Rosenquist et al. 1973) and human (Kuhn III 1968; Rosenquist 1981; Takaro et al. 1982, 1985; Sirianni et al. 2003, 2006; Roth-Kleiner et al. 2005; Behzad et al. 2009). These studies investigated alveolar (Hung et al. 1973) or smooth muscle cell (Campbell et al. 1978) innervation, connective tissue fibers (Rosenquist et al. 1973; Rosenquist 1981), alveolar epithelial cells with focus on AE1 (Mercurio and Rhodin 1976, 1978, 1984) or AE2 cells (Kuhn III 1968; Young et al. 1985), neuroepithelial bodies (Lauweryns and Cokelaere 1973; Rogers and Haller 1978, 1980; Pearsall et al. 1985), gaps in the alveolar capillary and epithelial basal lamina (Walker et al. 1995), linkage of alveolar capillaries and epithelium by fibroblasts in normal (Sirianni et al. 2003) or emphysematous lungs (Sirianni et al. 2006), the subepithelial fibroblast network in small human lung airways in controls and chronic obstructive pulmonary disease patients (Behzad et al. 2009), microvascular maturation (Burri and Tarek 1990; Roth-Kleiner et al. 2005) and interalveolar pores (Takaro et al. 1982, 1985; Weiss and Burri 1996).

These studies made use of an ample set of methodologies how, finally, 3D information can be gained and/or visualized and greatly enhanced our current 3D conception of different aspects on lung structure. The methods range from “simple” investigation of sequential images (Kuhn III 1968; Hung et al. 1973; Lauweryns and Cokelaere 1973; Rosenquist et al. 1973; Campbell et al. 1978; Rogers and Haller 1978, 1980; Rosenquist 1981; Takaro et al. 1985, 1982; Weiss and Burri 1996; Roth-Kleiner et al. 2005), over schematic 3D drawings (Rosenquist et al. 1973), “physical” modeling by stacking plastic acrylic (Mercurio and Rhodin 1976, 1978, 1984), acetate (Mercurio and Rhodin 1984), cardboard (Walker et al. 1995) or wood layers (Pearsall et al. 1985) to digital modeling by computer-assisted and computer-based reconstructions (Young et al. 1985; Burri and Tarek 1990; Sirianni et al. 2003, 2006; Behzad et al. 2009).

The informative value of serial sections and, thus, a 3D dataset, compared to non-serial or single images has been emphasized by different authors (e.g., Lauweryns and Cokelaere 1973; Rosenquist et al. 1973; Burri and Tarek 1990).

SsTEM, however, is both very labor intensive (Kremer et al. 2015) and technically challenging: Sections are susceptible to several artifacts like compression, folds, uneven thickness of the sections or the carrier film underneath, holes, cracks, dirt (see, Harris et al. 2006) (for compression in particular see, Sotelo 1957; Peachey 1958; Satir and Peachey 1958) and knife marks (see, Aescht et al. 2010). These may interfere with subsequent alignment of the dataset. Even worse than artifacts is that entire sections may become useless, for example, because of folds or film damage (Young et al. 1985) or may be lost completely (Rautiainen et al. 1984; see Saalfeld et al. 2012). The latter may result in the loss of important information necessary for correct structural interpretation or it may lead to a wrong “height” of the final model, if the loss is not corrected.

Correct interpretation of two-dimensional (2D) microscopic images requires knowledge of the correct scale (in the digital area, this is the pixel size of the images). This is achieved by proper calibration of the microscopic system. 3D reconstructions, however, are in addition strongly dependent on knowledge about the correct *z*-scale and, thus, on well-calibrated ultramicrotomes that provide sections of reliable thickness. One should be aware that setting the microtome does not per se lead to the desired section thickness (Young et al. 1985). Section thickness can be judged qualitatively by the section color on the water bath (Peachey 1958; Hoppert 2003) or be measured by different approaches: ellipsometry (Peachey 1958), measuring folds in the section (Young et al. 1985) or measuring the block before and after sectioning and calculation of the section thickness (Young et al. 1985). Advantages over the SEM techniques described below, are that ssTEM, as a TEM technique a priori provides a better *x*-, *y*-resolution and that it makes use of physical sections which can be post-stained to raise image contrast. Also specific labeling (for example, Hoppert 2003) as well as correlative (for example, Hegermann et al. 2019) approaches are possible. The preservation of ultrastructure can be improved using cryo-methods (see section “Electron tomography (ET)”). As a result, even fine subcellular structures can be analyzed. Furthermore, the method is the most modest in terms of equipment (Peddie and Collinson 2014) and the samples can be archived and reimaged; while in a serial block-face scanning electron microscopy (SBF-SEM) or focused ion beam scanning electron microscopy (FIB-SEM) study, the tissue under investigation will be lost forever, once the knife or gallium beam has unveiled the next plane for imaging.

With respect to the lung, no special demands are made on tissue preparation except that a postfixation step with half-saturated uranyl acetate (UA) is recommended to improve the preservation of surfactant and its intracellular storing organelle, the LB of AE2 cells (Fehrenbach et al. 1991). For review of surfactant see, Ochs and Weibel (2008) and for a detailed preparation protocol see, Fehrenbach and Ochs (1998). The uranyl cation reacts with phospholipid head groups (Ting-Beall 1980), and when omitted, the lipid lamellae are not well preserved, even in fresh material (personal experience). Furthermore, according to our experience, osmium tetroxide (OsO_4_) buffered in cacodylate instead of water and an additional infiltration step in 100% resin at 40 °C directly before the final polymerization improve LB preservation, while a gentle dehydration on ice and a dehydration start with 30% acetone have no major beneficial effect. So starting with 70% acetone at room temperature is more efficient. Also the treatment with propylene oxide shows no convincing benefit. An important factor, however, is in our opinion the time point of further sample processing after primary fixation. In studies—targeting at surfactant ultrastructure—samples should be embedded directly after fixation.

In contrast to other techniques, the volume for ssTEM analysis is in a way not restricted in *z*-dimension by the method, but only by the skill, motivation and effort of the investigator.

## Electron tomography (ET)

Electron tomography (ET) is a method for achieving 3D information with best resolution but in smallest volumes, compared to all other methods described here (McIntosh et al. 2005). ET differs from the other 3D methods in the way that virtual sections instead of physical sections are generated in silico from the sample material (i.e., a “thick” section), defining the method per se as “non-destructive”. The “thick” tomography sections (see below) containing the smaller structures of interest in their 3D context can be stored and used for ET analysis several times. The thicker the section, the more material is included for the tomographic investigation—however, also, more challenges arise during image acquisition (see below). Thus, for each question, the section thickness has to be adapted to the dimensions of the structures to be examined. Such “thick” sections cannot be properly depicted by 2D imaging, due to overprojection of all structures contained in the section.

The preparation of samples for ET can generally be performed in the same manner as for single or ssTEM: chemical or cryo-fixaton, labeling with specific markers or antibodies, dehydration and embedding at room temperature (RT) or by cryo-methods (Frank et al. 2002; Marco et al. 2004). Sections can be examined at RT from resin-embedded material or also as frozen hydrated sections at cryo-conditions in a TEM equipped with a cryo-stage (Al-Amoudi et al. 2004). Suspensions of particles smaller than a few hundred nanometers (viruses, purified organelles, macromolecules) can be mounted directly without sectioning either on a carbon film (at RT) (Hoppert 2003) or in a thin layer of vitrified water (at cryo-conditions) (Dubochet and McDowall 1981; Dubochet et al. 1982).

The principle of ET is comparable to computed tomography (CT) where an X-ray tube orbits around the body to apply radiation from different angles. The detected data are used for generating virtual sections. While for variation of the viewing angle in CT, the imaging device orbits around the patient, in ET, the object is tilted while the microscope and the camera are static.

In CT, images can be obtained from all angles in 360°; in ET, however, a certain range of viewing angles is impossible to reach. This is because tilting is limited to a maximum of approximately 70° in both directions from 0° as in higher angles the object retaining device, the bars of the EM grid, or the section itself will shadow the region of interest (ROI). As the viewing angles around 90° are missing, the method is “blind” for aspects oriented almost parallel to the section plane. This is referred to as the “missing wedge” (Saghi and Midgley 2012) or, when using multiple tilting directions, at least the “missing cone” (Neumüller 2018). Attempts have been made to overcome this using cylindrical holders (Palmer and Löwe 2014).

The achievable resolution of ET depends on the object and/or section thickness, the acceleration voltage used, the quality and calibration of the tilting device (goniometer), and also on the precise alignment of the resulting tilt series of images (Neumüller 2018). The signal-to-noise ratio (SNR), which generally suffers from inelastic scattering when using thick sections, is improved by high acceleration voltage and/or using energy filtering (Vanhecke et al. 2011). Tomography TEMs usually offer 200 or 300 kV. This permits a section thickness of up to ~ 300 nm. Thicker sections can generally also be used, but then the resolution declines (Ercius et al. 2015). Even sections of up to 1 µm thickness were used to analyze pulmonary capillaries using a 400 kV machine (West et al. 2010).

Due to this limitation of object thickness, ET is a method best suited for subcellular targets smaller than a few hundred nm, which are completely included in one section. Skilled people can succeed in recording and mounting serial tomograms from physical serial sections, and, thus, in obtaining ET reconstructions of thicker objects, for example complete cells (Höög et al. 2007; Weber et al. 2014; Mühlfeld et al. 2020). For such approaches, however, depending on the resolution required, FIB-SEM is often a more efficient solution.

ET and especially cryo-ET is a highly sophisticated method that is further developed, optimized and theoretically fully understood by few professional research groups (for reviews and some special procedures see, Al-Amoudi et al. 2004; McIntosh et al. 2005; Bonetta 2005; Lucić et al. 2005, 2008; Frey et al. 2006; Gan and Jensen 2012; Irobalieva et al. 2016; Castaño-Díez et al. 2017; Koning et al. 2018; Benjin and Ling 2020). Especially, the in silico processing of the acquired image tilt series is challenging. Kremer et al. (1996) connected physics and biology, by creating the IMOD software package, facilitating perfect alignment and reconstruction in ET.

Correlative approaches facilitate the determination of target regions using ssTEM (Popescu et al. 2011) and 3D light microscopy (Hegermann et al. 2019). Furthermore, the same lung tissue blocks embedded for ET can also be used for X-ray tomography to image much larger 3D volumes, though with less resolution, but containing numerous alveoli (Schittny 2018). ET is generally suited to investigate ultrastructural details that cannot be sufficiently resolved by SBF-SEM or FIB-SEM, for example mitochondria cristae (Frank et al. 2002), the ultrastructure of cilia and their cytoskeletal elements (Burgoyne et al. 2014; Lin et al. 2014), virus interactions with ciliated epithelia (Ke et al. 2018) or details of surfactant in tubular myelin or in LBs of AE2 cells (Ochs et al. 2016). ET could unveil the connection of a LB with an autophagosome, by revealing a common surrounding membrane in a tomogram (Mahavadi et al. 2015) (see Fig. 3).

Individual lamellae of LBs are almost impossible to resolve by SBF-SEM, as here high-contrast embedding protocols are needed (see “Serial block-face scanning electron microscopy (SBF-SEM)”), and LBs often get overstained. FIB-SEM does not necessarily require high-contrast embedding, but the single lamellae of LBs are at the edge of possible resolution and hard to discriminate, especially their curvatures and continuity. With classical embedding protocols (see “Serial sectioning transmission electron microscopy (ssTEM)”) using en bloc UA and one osmium step, individual intra- and extracellular surfactant lamellae are clearly discernible in a TEM on ultrathin sections (Ochs et al. 2020). ET also enables resolving the individual lamellae, even the two phospholipid leaflets (Ochs et al. 2016). Only cryo-EM and cryo-ET of frozen hydrated sections from vitrified material reveal unfixed lamellae properly without UA. However, for this approach, the lung tissue must be cryo-immobilized by high-pressure freezing (HPF), a method not compatible with air inclusions in the sample. To circumvent this limitation, the air can be replaced by a liquid in vivo (Vanhecke et al. 2010). By HPF, dynamic subcellular processes are stopped in milliseconds and preserved as close as possible to their living state, while during the much slower chemical fixation (at least several seconds), more severe alterations occur (McDonald and Auer 2006).

Membranes and also surfactant lamellae are best detectable when oriented vertically inside a section, both in 2D imaging of ultrathin sections as well as in ET of thick sections, in the latter, due to the “missing wedge” effect. For any structural component analyzed by ET, it is generally a prerequisite that it is silhouetted against the intra- or extracellular background by comparatively increased electron density.

ET will not shed light on the lung´s overall structure, but is the method of choice if best resolution in 3D is needed to analyze subcellular details that cannot be resolved by the other 3D methods.

## Serial block-face scanning electron microscopy (SBF-SEM)

In serial block-face scanning electron microscopy (SBF-SEM) an ultramicrotome is mounted in a SEM chamber. A diamond knife removes material of a resin-embedded sample and the SEM beam scans the exposed block-face. The first system was constructed by Leighton (1981). The breakthrough of this technique came several years later, when Denk and Horstmann (2004) rediscovered this method and optimized it with the latest technology, especially taking advantage of further developments in IT techniques.

The benefit of the block-face approach is that difficulties—due to handling of sections—are eradicated, such as distortions, compressions, damaging or losing sections (see “Serial sectioning transmission electron microscopy (ssTEM)”). Moreover, the whole process can run in a highly automated manner. Drawbacks are the reduced resolution of the SEM compared to (ss)TEM and the risk of charging issues due to the resin-embedded sample: Pure EM resins are generally insulators and only the organic material loaded with metal stains makes the sample sufficiently conductive. If the electron dose becomes too high, the resin gets damaged by the beam, leading to loss of resolution and diminished cutting properties of the sample. Especially in regions of empty resin, the lack of conductivity causes these problems. The lung with the “empty” airways and alveoli is in particular prone to these charging effects. For more details about SBF-SEM, the interested reader is referred to the comprehensive reviews of Kremer et al. (2015); Titze and Genoud (2016) and Smith and Starborg (2019), for example.

The SBF-SEM approach gives no opportunity to apply a post-staining as it is possible in ssTEM or array tomography (AT) analysis. Thus, the embedded samples need a priori good contrast and conductivity en bloc. These requirements are achieved by protocols (for example rOTO) introducing a lot of different metals like osmium, uranium, lanthanides and lead into the same sample (e.g., Seligman et al. 1966; Malick et al. 1975; Willingham and Rutherford 1984; Deerinck et al. 2010; Tapia et al. 2012; Hua et al. 2015; Odriozola et al. 2017). According to our experience, in particular, the alveolar septa of mouse lungs may become brittle during such a preparation and have to be handled with care (for example, a brush can be helpful to transfer samples during preparation).

The advantage of lung samples, however, is that they are well infiltrated by chemicals because of the easy access of fluids to the delicate tissue (after a deep inspiration more than 80% of the lung volume consists of air (see, Knudsen and Ochs 2018)). This means that long infiltration times are not necessary and problems with staining gradients are very rare. For more challenging samples or large sample sizes, the BROPA (Mikula and Denk 2015) and fBROPA (Genoud et al. 2018) protocols, which focus on homogenous staining and infiltration, may be beneficial.

To reduce charging effects, some groups optimized preparation protocols using resins containing carbon (Nguyen et al. 2016) or silver (Wanner et al. 2016) to tightly encase the samples and increase conductivity. However, an infiltration of tissues with a conductive resin has (according to our knowledge) not been reported yet.

For the preparation of lung samples, it seems reasonable to use a filler for the airways to reduce regions with empty resin. Applying agar or agarose into the lung is well established (for example, Callas 1974). Also gelatine (Sanderson 2011) or bovine serum albumin (BSA) (McDonald et al. 2007) are known as extracellular filler or cryoprotectant in preparation techniques. According to our experience, low-melting point agarose improves the conductivity better than BSA or gelatine. However, the benefits are minimal, so we prefer using the variable pressure mode (see below) instead of fillers. Of course, it is an option if nuances make the difference.

In variable pressure mode, nitrogen gas is passed into the chamber to compensate charging of materials. This enables scanning of poorly conducting samples and protects the resin to a certain extent from beam damage. Unfortunately, this happens at expense of resolution due to interaction of gas molecules with the electron beam. According to our experience, the variable pressure mode is the best option for fast imaging of large ROIs and also reduces the risk of section debris falling back onto the block-face.

An advanced development was presented by Deerinck et al. (2018) with focal gas injection-based charge compensation. Here, the nitrogen gas is applied with a needle in direct proximity to the block-face. Thus, the SEM can work in a range of high vacuum with good resolution and SNR, while the sample block-face profits from the charge compensation. This approach is probably the most promising technique to deal with the pitfalls of lung samples.

Compared to the other volume EM techniques presented here, the fields of application of SBF-SEM analysis are in most cases “large” volumes (with respect to EM dimensions), in our hands mostly volumes up to 500 µm in *x*-, *y*- and *z*-dimensions. Figure 5 illustrates some important parameters which have to be considered for each research question: The sample should be as small as possible to achieve the best conductivity and cutting properties. The dwell time (scan time per pixel), the acceleration voltage, the beam current and the pixel size influence the SNR. The resulting dose determines the risk of beam damage of the resin and/or charging artifacts. Imaging parameters have to be tested for each project over several sections with respect to adequate resolution to make sure that the sample remains stable with the chosen section thickness. Besides the scanning parameters for adequate imaging, the resulting total recording time and data size have to be considered (this is true for all volume SEM techniques, see Fig. 5). Thus, the crucial question for each acquisition is which details of the ultrastructure have to be discernible (also in *z*-dimension), and what is irrelevant for a specific scientific question. One must be aware, that the recording of a single image stack can take several months and data storage may require several terabytes (see, Titze and Genoud 2016). One strategy could be to work with different ROIs (also known as multi-ROI). For example, the whole block-face is imaged with low resolution as survey and special events occurring over time in *z*-direction are scanned as additional, separate ROIs with higher resolution.

To compare different imaging requirements, for example, the following two studies can be considered: Schneider et al. (2019) needed the high resolution provided by high vacuum to identify AE1 cell borders; whereas, Buchacker et al. (2019) needed a larger field of view (FOV) but could abdicate highest resolution to analyze the alveolar capillary network in mice. Further applications of SBF-SEM in lung research were a study of West et al. (2010) exploring blood and air capillaries in the chicken lung and imaging of immune cells in the mouse lung by Kremer et al. (2015).

## Focused ion beam scanning electron microscopy (FIB-SEM)

Focused ion beam scanning electron microscopy (FIB-SEM) also enables the production of 3D models based on serial block-face images. Similar to SBF-SEM, material is consecutively removed from resin-embedded tissue inside a SEM chamber. Unlike SBF-SEM, the material is not removed by a diamond knife, but by a focused ion beam (FIB). The FIB-SEM is equipped with two emission sources: One conventional SEM column on top producing the electron beam for imaging and a second column aside, emitting an ion beam (in most applications consisting of gallium ions), which is focused onto the sample surface for abrasion of sample material. The strength of the ion beam is variable, thus enabling rough trimming of the ROI (high beam current), and fine milling of the final FOV (low beam current). The two beams (electrons and ions) are focused onto the same sample area and work together automatically by consecutive/continuous imaging and milling, and, thus, create stacks of serial images (for details see, Knott et al. 2011; Kizilyaprak et al. 2014, 2019; Narayan and Subramaniam 2015). The fine ion beam can mill layers below 5 nm thickness, in contrast to the diamond knife used in SBF-SEM, which cuts at least 50 nm, in certain cases thinner depending on the sample (personal experience). The *z*-resolution is, therefore, much higher in FIB-SEM. In exchange, the achievable volume is much smaller than in SBF-SEM, as the ion beam can mill only some tens of µm in *x*-, *y*-direction, while the diamond knife is able to cut larger areas (see above).

A recent study on the human alveolar epithelium demonstrates the benefit of FIB-SEM for the visualization of fine structural details: FIB-SEM could image an almost complete AE2 cell and enable detailed reconstructions of a LB secretion event, the microvilli lawn on the cell surface and tiny AE1 cell processes beneath AE2 cells (Schneider et al. 2020). Also subcellular details in AE2 cells (plate bodies) could be revealed in 3D using FIB-SEM (Mühlfeld et al. 2020).

FIB-SEM has the great advantage over SBF-SEM to be applicable on archive material conventionally embedded for TEM, as high-contrast achieving protocols like rOTO (see above) are not required (Hegermann et al. 2019; Steyer et al. 2020; Wrede et al. 2020). Similar to SBF-SEM, however, the resin block surface is made conductive by coating it with metals and carbon (Luckner and Wanner 2018; Wrede et al. 2020), but while in SBF-SEM this coat is removed by the diamond knife, in a FIB-SEM, the resin block stays coated and only the small FOV of a few hundred µm^2^ is unsealed by the FIB. In this way, charging effects are minimized, making imaging much more feasible compared to SBF-SEM.

Another advantage of FIB-SEM versus techniques based on cutting with diamond knives (ssTEM or conventional TEM, SBF-SEM, AT) is that the FIB can cut any material that might cause problems if cut with a diamond knife because of its hardness or instable embedding in the resin, both possibly causing damage to the diamond knife or loss of material of interest during the cutting process. Carbon nanotubes, for example, are impossible to be cut for TEM or SBF-SEM, while the FIB cuts them easily and facilitates their detection and 3D reconstruction in lung tissue (Købler et al. 2014).

FIB-SEM is, as the only SEM volume technique, also applicable under cryo-conditions, using HPF sample material, which provides best structural preservation, though the contrast is lower than in embedded samples (Schertel et al. 2013). A FIB can, as an alternative to classical ultramicrotomy, also be used to prepare thin lamellae of any material for further imaging in a TEM (Giannuzzi and Stevie 1999). As mentioned, the cutting capability of the FIB is not restricted by the material, which is especially a benefit when working in cryo-conditions (Rigort and Plitzko 2015; Zachs et al. 2020): Cryo-sectioning with a microtome comprises multiple challenges concerning compression and crevasses, the latter become more severe with increasing section thickness above 100 nm (Al-Amoudi et al. 2005). Using a FIB, also thicker lamellae can be prepared without the mentioned artifacts and cryo-transferred into a TEM (Wagner et al. 2020), which may be desirable for cryo-ET for reconstructing volumes thicker than 100 nm. LBs of AE2 cells could be imaged and reconstructed in a volume of 200 nm thickness by this procedure in cell culture (Klein et al. 2020), which can be vitrified much better than native lung tissue (see above).

The achievable *x*-, *y*-resolution in FIB-SEM, as determined not by theoretical calculation but by judging what we see, comes close to TEM: membranes are visible and in high magnifications, it may even be possible (by spending effort on optimizing focus and stigmation), to get a glimpse of the phospholipid bilayer (Heymann et al. 2009). Using HPF/freeze substitution with high contrast achieving protocols in combination with recording the secondary electron signal from the block surface, can even improve the membrane contrast, getting even closer to TEM quality (Villinger et al. 2012). In lung tissue, however, the use of HPF is limited (see “Electron tomography (ET)”). LBs can also be imaged using FIB-SEM in critical point dried samples by presenting an intersection of the interior after abrasion of the sample surface by the FIB, though with less preservation of the ultrastructure compared to embedded samples (Drobne et al. 2008).

FIB SEM bridges a gap between SBF-SEM and TEM by providing a resolution close to TEM, though with smaller volumes than possible in SBF-SEM.

## Array tomography (AT)

Array tomography, including ATUM-SEM (see below), is basically very similar to ssTEM with the essential difference that serial sections are imaged with a SEM instead of a TEM. In contrast to ssTEM, the carrier is not as much limited in the amount of space for sections as a TEM grid is. Carriers are often tapes, silicon wafers, microscopy slides or coverslips, also as conductively coated versions, for example with indium tin oxide (ITO) (Wacker and Schröder 2013; Baena et al. 2019). Using these substrates, it is easily possible to collect a higher number of sections compared to using grids for ssTEM. The latter offer only a few mm^2^ for the sections. In case of collecting sections on tape, thousands of sections are possible in a highly automated manner. This approach was significantly developed by Hayworth et al. (2006) and is known as Automated Tape Collecting Ultramicrotome SEM (ATUM-SEM) (Schalek et al. 2011, 2012) in the latest version. Here, comparable to TEM sectioning, a diamond knife in a conventional ultramicrotome cuts sections. These sections float on a water-filled knife boat and are collected by a moving tape which is subsequently reeled up (similar to an audio tape). The sections are usually quite large for ultramicrotomy (they can be larger than a TEM grid) and are collected consecutively as separated sections with the tape. Nevertheless, also contiguous ribbons of sections can be picked up as packets by the tape. To image the sections, the tape is cut into stripes and mounted with double-sided conductive adhesive tape onto silicon wafers (Baena et al. 2019).

A frequently used tape is Kapton (Baena et al. 2019), a very stable polyimide, but with the disadvantage of being a hydrophobic insulator. Plasma glow discharge can be used to make the tape more hydrophilic and improve the section pick up without artifacts like wrinkles. Carbon coating of the tape and/or use of conductive silver paint/copper tape after mounting can improve conductivity and, thus, imaging conditions (Hayworth et al. 2014; Baena et al. 2019). Recently, very promising results to further minimize charging issues have been shown with a conductive carbon nanotube (CNT)-coated polyethylene terephthalate (PET) tape (Kubota et al. 2018).

Under conditions without ATUM, a large diamond knife boat enables the transfer of long section ribbons on microscopy slides or silicon wafers, for example (Micheva and Smith 2007). Here, in addition to pure manual handling, a substrate holder (Wacker et al. 2016, 2018) can be used to facilitate serial sectioning for AT. Similar to ssTEM, it must be taken into account that serial sectioning is demanding and needs experience in ultramicrotome operation, and, thus, for AT, auxiliary equipment like the substrate holder may be useful to minimize artifacts. Here, in contrast to the ATUM technique, contiguous ribbons of sections are beneficial to get a high number of sections onto the substrate.

An essential advantage of these array techniques in comparison to the block-face techniques is that it is non-destructive. In contrast to SBF- and FIB-SEM, the material is not lost. This opens up a number of new opportunities. The sections can be imaged several times, for example with sequentially higher resolutions and adapted ROI size, cf. Figure 2. The advantage, in particular if rare events are investigated, lies at hand: After having imaged the entire volume with low resolution for orientation, a specific ROI with the desired rare structure of interest can be reimaged again with high resolution. A possible scenario in the lung may be the investigation of the composition of an entire alveolus where one needs to start imaging at its cap, or the investigation of the bronchioalveolar duct junction which serves as a stem cell niche (Schittny 2017). In block-face techniques, where also high- and low-resolution ROIs are possible, the first occurrence of the desired structure has to be recognized during the dataset acquisition just in time and even then, the first nanometers have already been lost. Therefore, it is more difficult in block-face techniques to target a structure and image it completely.

Sections for AT are best stored under vacuum (personal experience). Moreover, it has to be noted that the electron beam, depending on the dose, can also damage the sample, and this may prevent further imaging. Accordingly, unlimited re-recording of a distinct area is not feasible.

A further advantage of AT is the possibility to apply a post-staining (Kasthuri et al. 2015) to the sections to enhance the signal or even—if appropriately fixed and embedded—to perform an immuno-labeling. Also, a correlative workflow with light microscopy (including fluorescence) is possible (Smith 2018). For the combination with light microscopy, a transparent carrier or tape is necessary; while for fluorescence analysis, the substrate should be non-autofluorescent, both provided by the CNT tape (Kubota et al. 2018).

From all volume EM techniques discussed here, AT offers to image the largest FOV, even in high resolution, only limited by the diamond blade and the cutting properties. These large areas can be recorded with many overlapping tiles (like a mosaic) (cf. Takaro et al. 1985 who applied montages in ssTEM) in a highly automated manner. By new developments, like a multi-beam SEM (Eberle et al. 2015) with several simultaneous beams, projects with previously impossible dimensions in combination with high resolutions become feasible since the scanning time is reduced a lot. Because of increasing data volumes, such applications raise new challenges for post-processing as well as data evaluation and storage.

On the contrary, AT promotes not only large volumes with more data, but also enables—as a versatile tool—to reduce the data volume with fast and efficient workflows by targeted approaches. For instance, AT permits the use of semithin sections (~ 200–300 nm) for fast light microscopical screening along the *z*-axis on several microscopy slides. Thus, distinct events can be identified in the tissue and subsequently imaged (using the same sections) with higher resolution by the SEM, as applied by Beike et al. (2019) who investigated AE2 cells in a mouse model of pulmonary fibrosis (cf. Fig. 2) or in a similar approach by Kataoka et al. (2019) who investigated lung autopsy specimen after influenza virus infection.

Drawbacks of AT, other than the aforementioned charging issues, are similar to ssTEM and have already been discussed there, for instance artifacts like compression, folds, knife marks and the risk of losing individual sections completely. These particular problems, however, are less severe in AT because of larger carriers and the possibility of a more automated section pick up (ATUM/substrate holder). However, charging and beam damage have to be taken into account. The sections, even if arranged in perfect ribbons, are never absolutely in line. Therefore, the post-processing, in particular the alignment procedure, is much more challenging than in block-face techniques (this is also true for ssTEM).

## From volume EM datasets to 3D analysis and models

Computer-assisted 3D reconstructions from EM serial sections have been carried out at least as early as the beginning 1970s (Levinthal and Ware 1972; Lopresti et al. 1973; Macagno et al. 1973; Levinthal et al. 1974). Nowadays, in our digital era, they became standard and are carried out using sophisticated image processing and 3D reconstruction software. It is beyond the scope of this review to cover the full range of various software packages. Instead, we will focus on programs that have turned out to be well suited for our 3D studies and are all freely available; a compilation of more software packages is found in the review of Borrett and Hughes (2016). Especially with several different users it is beneficial to work with freely available software. As analysis frequently takes longer than the initial data acquisition, this overcomes a critical bottleneck in the workflow, because researchers can use their own IT capacities. The general workflow for preparing a 3D analysis of the structure(s) of interest can be subdivided into three distinct steps:

1. Image acquisition (i.e., generation of a raw dataset)
2. Post-processing of the raw dataset (i.e., making the dataset usable)
3. Investigation, segmentation and modeling (i.e., performing the actual analysis)

It should be emphasized here, that the image acquisition by one of the volume EM techniques is only the beginning of the 3D study. All techniques have in common that they only provide a raw dataset of incremental *z*-planes constituting the sample volume. These *z*-planes contain incremental profiles of the structures of interest, which can be used for “reassembling” and exploring their 3D structure. To obtain a first overview of the dataset, a down-scaling (binning) to about 1–2 k pixels edge length and 8 bit can be useful (see below). Another option is the image pyramid function of the IMOD package (Kremer et al. 1996), which generates a set of files that enables rapid reading of the dataset and fast navigation through it at different resolutions with standard IT hardware, similar to navigation software. Also, Fiji (Schindelin et al. 2012) offers a similar option with the BigDataViewer (Pietzsch et al. 2015).

Several post-processing steps, requiring time and IT resources, may be necessary before the actual 3D analysis can start. These may include normalization and/or inversion of gray values, reduction of the dataset to a manageable data volume with respect to hardware resources (e.g., by conversion from 16 to 8 bit, down-scaling (i.e., binning) of the dataset, cropping of the dataset to the region (volume) of interest), application of filters or image alignment (cf. Schneider et al. 2019). As output, a compatible file format that enables lossless image storage should be used (for example TIFF). Especially for large datasets, it may be easier to work with single image files instead of one file containing all images, since some programs cannot process such large data files. Fiji (Schindelin et al. 2012), a distribution of ImageJ (Schindelin et al. 2015), is a comprehensive open source image processing software that covers all of these functions and is, therefore, often used for our post-processing. The Microscopy Image Browser (MIB) (Belevich et al. 2016) may serve as good alternative or complementary software. This software package is tailor-made for microscopy applications with a focus on 3D data stacks. Amongst others, it comprises image processing as well as versatile segmentation tools.

Proper image alignment is an important prerequisite for the 3D analysis. It means that the images have to be arranged in a way so that the pile restores the original volume (cf. Saalfeld et al. 2012). An aligned dataset can be viewed as a smooth movie (Levinthal and Ware 1972; Levinthal et al. 1974), where one gets the idea of “walking through the structure” (Levinthal et al. 1974). The alignment influences the “easiness” (and, thus, efficiency) of the tedious modeling process: The better the alignment, the easier the structures can be followed by eye in the *z*-direction for investigation or modeling and the better the esthetics (smooth surface) of the final model. Eventually, it may be necessary to remove and replace images/sections with severe artifacts that might prevent proper alignment (see, Schneider et al. 2019). This is in particular relevant for techniques susceptible for such artifacts, like ssTEM, AT and SBF-SEM in high vacuum. In the latter, it may happen that former sections fall on the surface to be scanned and occupy landmarks for proper alignment. Some alignment tools (like the elastic alignment (Saalfeld et al. 2012)) can deal with this, because they can consider more than only one adjacent image, while others may abort or fail with the alignment process. Besides using automated algorithms, alignment can also be done completely manually. This may be necessary to handle difficult artifacts like large folds known from ssTEM or big shifts (for example, after ROI adjustment or for joining stacks).

Available alignment tools range from mere translation, over rotation and scaling (methods that do not alter proportions) to methods that include transformations aiming at reverting deformations caused by the section/image acquisition. This range of methods is satisfactorily covered by Fiji including its plugin TrakEM2 (Cardona et al. 2012) and MIB. Simple tools for checking the “quality” of the restored volume are thorough investigation (looking for jumps or odd distortions) of the aligned dataset along z and the investigation of lateral views (cf. Huijsmans et al. 1986). If alignment methods that deform images are applied (like elastic alignment (Saalfeld et al. 2012)), we recommend to compare this alignment with an alignment method that only uses translation and (if necessary) rotation (because the deforming alignment itself may cause artifacts). Some authors (Huijsmans et al. 1986), in general, even demand to include realignment marks before slicing, because “otherwise the implicit but broken coherence between sections can only be restored on a visual (subjective) basis” (Huijsmans et al. 1986). Block edges or tracking/focus marks in the deposition layer of FIB stacks can be used as reliable marks for the alignment. A brief overview of useful post-processing steps is given in Table 2.

**Table 2:** Overview of useful post-processing steps. For more details and the practical application, the reader is referred to the documentation of appropriate imaging software like Fiji (https://imagej.nih.gov/ij/docs/guide/user-guide.pdf [accessed July 8th, 2020]), or Microscopy Image Browser (https://mib.helsinki.fi/documentation.html) [accessed July 16th, 2020])

Removal of artificial images/ correction for lost images	Severe artifacts may interfere with alignment and thus with subsequent 3D analysis. Such images are removed. To not modify the *z*-dimensions of the dataset, the previous or next image is doubled. Also, lost images can be replaced in this way to keep the *z*-dimension of the stack (cf. Borrett and Hughes 2016)
Conversion to 8 bit	Commonly, 8-bit grayscale images are used (i.e., 2^8^ = 256 different gray values can be assigned to a single pixel) (Wolf 2013). In our opinion, this is sufficient for human eye investigation and manual segmentation. Some imaging devices, however, may output images of more than 8 bit (e.g., 16 bit). Conversion of these images to 8 bit considerably reduces the size of the dataset
Inversion of gray values	Some imaging devices may output images with inverted grayscales (compared to conventional TEM images). To get the familiar TEM appearance with dark tissue and bright empty resin grayscale inversion may be necessary. Alternatively, the LUT (Look Up Table) may be inverted
Cropping	Volume EM data acquisition may result in a big surprise. In particular when SBF-SEM or FIB-SEM is used, one often does not know what the final dataset will contain, when the acquisition is started. At best, one gets a dataset that includes the desired structures of interest completely—the larger the imaged volume the higher the chance of complete structure(s) of interest. Subsequent cropping reduces the “biological” volume and, thus, the hardware demanding data volume. “Cropping” in the *z*-direction is achieved by simply using the images of the desired image range. Cropping in the *x*- and *y*-direction is achieved by cropping the images. Care has to be taken, that the structures of interest are not cut off, since they may “move” out of the selected region for cropping
Alignment	Alignment is necessary for restoring the original volume before the sample was sectioned. For more details, see text
Binning	Binning compresses the dataset by converting several pixels into one. This can be applied along the *x*-, *y*- and *z*-axis. As a consequence, the pixel size increases which may impede proper recognition of the structures to be analyzed. Thus, the level of binning has to be pondered carefully
Filtering	The application of filters on the dataset may facilitate the later 3D study. Mean or median filters, also in 3D, for example, may reduce image noise
Normalization	Varying gray value intensities resulting in a gradient along the *z*-stack can be harmonized to give the dataset a consistent grayscale distribution. MIB has proven to be a useful tool for this step. In case of problems due to surrounding structures, a mask can be applied to normalize just the targeted volume. Contrast-limited adaptive histogram equalization (CLAHE) (Pizer et al. 1987) can be beneficial for gradients within single images

Because of the tremendous amount of data (up to several terabytes) (see, Titze and Genoud 2016), the post-processing makes some demands on time and computer hardware. According to our experience the most important resources are storing capacities (raw data, processed data, backups), memory and a solid state disk (SSD). Indeed, Fiji is able to open the dataset as virtual stack, i. e., only the relevant images are loaded and processed to save resources, but to view the dataset fluently, the memory should exceed the data amount to be viewed. A SSD considerably reduces the time for loading the dataset.

Finally, after weeks to months of tissue preparation, image acquisition and post-processing (depending on the methods applied and the imaged volume) the resulting dataset can be used for 3D analysis. The structures of interest can be investigated in a smooth movie of the dataset (see above), in different section planes or be reconstructed in 3D. A global “overview” model can be provided by UCSF-chimera (Pettersen et al. 2004), based on gray value thresholding. Specific gray values can be displayed and assigned to colors. In combination with the options of cropping and transparency distinct structures can be visualized in 3D. This model can later be combined with other segmentation-based reconstructions (see below).

A well-established program for detailed 3D reconstructions in a *z*-stack of images is 3dmod of the IMOD package (Kremer et al. 1996). It is stable as well as well documented and maintained by its developers: After import of images, objects are defined and their profiles are delineated on different section planes by manually tracing their boundaries. The resulting pile of contours is then utilized by 3dmod for modeling the structures (cf. Schneider et al. 2019, 2020). Among other things, neighboring contours (below or above the current image), several planes at a time or arbitrary section planes can be displayed. The model can be rotated and individual objects (e.g., individual cells) can be shown or hidden, or made transparent. Furthermore, it has a clever option for preparing smooth movies. The basic workflow of creating 3D models in 3dmod and some visualization options are illustrated in the accompanying video of (Schneider et al. 2019), available under https://www.youtube.com/watch?v=-iKowVFGKgY [accessed June 06, 2020].

MIB may serve as a valuable alternative software package with a well-organized user interface and video tutorials. It offers versatile segmentation tools supporting the user not only with manual but also semi-automatic functions, including a good line and shape interpolation. With a brush tool, for example, superpixels, based on simple linear iterative clustering (SLIC) (Achanta et al. 2012) or watershed algorithm, can be used to rapidly segment biological structures. Using erode or dilate options, the segmented area can be easily fitted. By means of global, local or adaptive thresholding, desired grayscales can be recognized and segmented. Besides an integrated visualization option, the models can also be exported to other programs like IMOD, Chimera, 3D Slicer (Fedorov et al. 2012; Kikinis et al. 2014) and others.

Depending on the structure of interest, it may be challenging to identify the outlines of a particular profile on a particular image with satisfying confidence. Valuable tools in such situations are: watching consecutive images in a fast progression back and forth, investigating the uncompressed images with higher resolution, applying different filters like mean or median filters in 2D or 3D to improve the SNR, adjusting brightness or contrast, preparing a substack of only the challenging region with subsequent anew alignment, investigating orthogonal or arbitrary planes, etc. (cf. Schneider et al. 2019). Having Fiji, 3dmod and MIB, all of these tools are available. Besides manual segmentation, also automated segmentation by image analysis is nowadays possible. This topic, however, is beyond the scope of this review.

Manual segmentation is a laborious work, but it reimburses the investigator in different ways. For example, artifacts can be recognized by the experienced investigator and treated as such, but in particular, manual segmentation inevitably gives the investigator insights into the entire volume and the interstructural relationships of objects which will not be detected if automated algorithms by image analysis are applied and only the final models of specific objects are investigated.

## Concluding remarks

EM enables the investigation of cells and tissues at the nanometer scale. Since its beginning it has strongly increased our knowledge about our body’s ultrastructure. Although the new advanced light microscopic techniques known as “super-resolution” microscopy (e.g., STED, PALM or STORM, (for review, see Vangindertael et al. 2018)) are able to provide a much better resolution than conventional light microscopy and have even entered the range of EM, they have the drawback that they need fluorescence as basic principle and offer no “open view” on all abundant ultrastructural details—expected or unexpected—as electron microscopy does (Ochs et al. 2016) (see also, Gopal et al. 2019).

With volume EM techniques, this open view is extended to the third dimension (Ochs et al. 2016), which is invaluable for understanding the 3D ultrastructure of our body’s organs such as the lung. In particular, the gain of topographic and relative information about the different tissue constituents, which may be of great functional relevance for their interplay, has to be emphasized (cf. Pearsall et al. 1985; Takaro et al. 1985). Conventional SEMs can reveal cellular surface details but cannot look into the cell simultaneously. (Single section) TEM reveals intracellular details, but may fail to transport important topographic information like the belonging of certain profiles (see, Rogers and Haller 1978; Schneider et al. 2019, 2020). The 3D information gained by volume EM, however, allows scientists to see arbitrary section planes in EM resolution and to generate 3D models that can be viewed from any angle with variable transparency. This enables a topographic knowledge gain that is not possible with single 2D images alone. Since the “new” and (automated) volume EM techniques like SBF-SEM, FIB-SEM and AT enter more and more the EM laboratories around the world, probably an extensive morphological reevaluation of our body’s ultrastructure will take place in the next years. Because of its complex ultrastructure with branching alveolar epithelial cells serving more than one alveolar surface, the extraordinary fine alveolar capillary network (“sheet flow” concept), a continuous and directed connective tissue network, etc. (see, Ochs and Weibel 2008), the lung is an organ that may notably benefit from these techniques. Some studies have already made use of them successfully (e.g., Schneider et al. 2018, 2019; Beike et al. 2019; Buchacker et al. 2019; Mühlfeld et al. 2020) and certainly, there are many more to come. They may significantly enhance our comprehension of the lung’s complex microanatomy, shed more light on known and so far unknown structure–function relationships, both under (physiological) mature, developmental and aging conditions, and patho(physio)logic conditions like lung injury and repair (morphological reconstitution of the blood–gas barrier) (cf. Ochs et al. 2016).

With respect to structure–function relationships, it will be interesting to combine some of the new volume EM techniques with immuno-labeling (see, Schroeder-Reiter et al. 2009; Boey et al. 2019; Gopal et al. 2019). In this way, molecular information is provided in the full 3D context and analyses of the spatial distribution of certain antigens or their preferred association with specific structural elements become possible; certain functions may, thus, be linked directly to these elements (Gopal et al. 2019).

The possibility to obtain also quantitative data—either based on segmentations (e.g., Young et al. 1985) or on stereology (as discussed by Ochs et al. (2016) and Mühlfeld et al. (2018))—as well as the higher chance of imaging rare events such as exocytosis (Young et al. 1985) further emphasize the value of volume EM.
